# Effectiveness of enhanced check during acute phase to reduce central venous catheters-associated bloodstream infections: a before-after, real-world study

**DOI:** 10.1186/s13756-022-01190-z

**Published:** 2022-12-06

**Authors:** Yu Lv, Xiaobo Huang, Qian Xiang, Qin Yang, Jin Chen, Minhong Cai, Pingping Wang, Ping Jia, Hui Wang, Caixia Xie, Luting Li, Dingding Zhang, Daoqiong Wei, Jiayu Wu

**Affiliations:** 1 Healthcare-Associated Infection Control Center, Sichuan Academy of Medical Sciences, Sichuan People’s Hospital, School of Medicine, University of Electronic Science and Technology of China, Chengdu, 610072 Sichuan People’s Republic of China; 2 Intensive Care Unit, Sichuan Academy of Medical Sciences, Sichuan People’s Hospital, School of Medicine, University of Electronic Science and Technology of China, Chengdu, 610072 Sichuan People’s Republic of China; 3 Department of Nursing, Sichuan Academy of Medical Sciences, Sichuan People’s Hospital, School of Medicine, University of Electronic Science and Technology of China, Chengdu, 610072 Sichuan People’s Republic of China; 4 Development Department, Chengdu Yiou Technology Co. LTD, Chengdu, 610000 Sichuan People’s Republic of China; 5 Sichuan Provincial Key Laboratory for Disease Gene Study, Sichuan Academy of Medical Sciences, Sichuan People’s Hospital, School of Medicine, University of Electronic Science and Technology of China, Chengdu, 610072 Sichuan People’s Republic of China

**Keywords:** Central venous catheters associated bloodstream infections, Insertion checklist, Maintenance checklist, Acute phase

## Abstract

**Background:**

To evaluate the effectiveness of enhanced check to the duration of the central venous catheters associated bloodstream infections (CABSIs), and the impact on infection rates.

**Methods:**

A before-after, real-world study in six adult intensive care units was conducted. All adult patients who had only one central venous catheter were included during two consecutive periods. The intervention period, added cross-check that all patients with central venous catheter (CVC) need to be performed, and included nurses' checks for insertion practices and doctors' checks for maintenance practices. Propensity scores matching were used to account for potential confounding, and restricted cubic spline was served as visualizing the CABSI risk.

**Results:**

A total of 2906 patients with 26,157 CVC-days were analyzed. After intervention, the density incidence of CABSI decreased from 10.24 to 6.33/1,000 CVC-days (*P* < 0.001), and the acute period of rapid increase in CABSI risk was shortened, 6.5 to 5 days for femoral-vein catheterization and 7 to 5.5 days for subclavian-vein catheterization. For jugular-vein catheterization, the acute onset period disappeared.

**Conclusion:**

Enhanced check during the first 7 calendar days after CVC insertion shortens the duration of the CABSI acute phase and tends to decrease CABSI rate.

**Supplementary Information:**

The online version contains supplementary material available at 10.1186/s13756-022-01190-z.

## Introduction

Central venous catheters (CVCs) are indispensable in the modern-day intensive care of critically ill patients [1]. However, this procedure can cause sometimes life-threatening complications, including CVC-associated bloodstream infections (CABSIs), which result in considerable morbidity, mortality, prolonged length of stay (LOS) and excess hospital costs [2, 3]. Reported rates of CABSI range from 1.2–46.3 per 1000 catheter-days on adult intensive care units (ICUs). The high baseline incidence of CABSI was considered to be associated with geographical distribution [4]. The International Nosocomial Infection Control Consortium (INICC) stated that the pooled incidence of CABSI in Asia was nearly five times higher than that reported from comparable ICUs in the United States of America (USA) [5]. Furthermore, the report from China National Clinical Improvement System (NCIS) showed that the incidence of CABSI had not decreased significantly in recent years [6]. Efforts to decline the incidence of CABSI are paramount in China.

The duration of catheterization was also considered to be clearly associated with the risk of CABSI [7, 8]. At present, most randomized controlled trials and practice guidelines focus on the effect of scheduled catheter replacement, but ignore the fluctuation of CABSI risk with the length of dwell time, which may provide a window of opportunity for infection prevention and control (IPC) [9–12]. In our previous baseline data analysis, it was stated that the first one week after CVC insertion was the acute phase of CABSI [Reference: Lv Y, Huang X, Lan Y, et al. Peripherally inserted central catheters have a protective role and the effect of fluctuation curve feature in the risk of bloodstream infection compared with central venous catheters: a propensity-adjusted analysis. BMC Infect Dis. 2022;22(1):289. 10.1186/s12879-022-07265-x]. This period was considered to be an important opportunity and substantial benefits could be obtained if the guidelines were well followed during this period [13]. Despite the evidence-based guidelines, a substantial implementation gap still exist between best evidence and best practice [14]. Creating independent redundancies, through the use of a checklist, was considered to be an effective technique to ensure physician and nurse adherence to best practice [15]. We hypothesized that the best practices in the acute phase were associated with the reduction in CABSI-incidence. This study aimed to explore the effectiveness of enhanced check during acute phase on reducing CABSI-incidence.

## Material and methods

### Study design

We did a prospective, before–after, real-world study in the six adult ICUs (Surgical-ICU with 36 beds, Emergency-ICU with 26 beds, Neurosurgical-ICU with 21 beds, Neurology-ICU with 13 beds, Geriatrics-ICU with 20 beds and Medicine-ICU with 19 beds) of Sichuan Academy of Medical Sciences, Sichuan People's Hospital, School of Medicine, University of Electronic Science and Technology of China, a 4200-bed tertiary care teaching hospital in Chengdu in the region of Sichuan (Western China). All adult (≥ 18 years) patients with only one CVC insertion admitted during January 2019 to December 2021 were included, avoiding the possible biases caused by the interaction between different catheter insertion states, such as insertion time and insertion sites. Patients hospitalized ≤ 2 days and/or catheterized ≤ 2 days were excluded. After matching screening, the remaining participants were included in the final comparative analysis. The informed consent was waived by the local ethics committee as it was an observational study.

### Study protocol

Two consecutive periods (Period 1, from January 2019 to December 2020, and Period 2, from January 2021 to December 2021) were set. Period 1, the control period, included a basic combination of prevention bundle for CVC insertion based on the published best practices, and a insertion checklist (Additional file 1: Table1) completed once a week by the part-time IPC professionals who has received unified education and has been authorized by the infection management department to stop the procedure if breaches in aseptic technique are observed. Major components of bundles included insertion qualification; hand hygiene; daily skin cleaning with 2% chlorhexidine; use of masks, caps, sterile gloves, sterile gowns, full-body sterile drapes, and the correct dressings; site disinfection with an alcoholic chlorhexidine solution containing more than 0.5% chlorhexidine gluconate (CHG) for CVC insertion; and use of the ultrasound guidance for internal jugular (IJ) catheter insertion.

Period 2, the intervention period, added cross-check and included two processes that all ICU patients with CVC insertion need to be performed: Nurses' checks for insertion practices performed by doctors, and doctors' checks for maintenance practices performed by nurses. To effectively deal with the acute phase of CABSI within 7 days after CVC insertion, we developed another standardized checklist (Additional file 1: Table 2) to be completed by the physician. To help ensure enforce infection control practices for CVC maintenance, three checks were carried out within 7 days after CVC insertion, and the time point were the first day, the fourth day and the seventh day after CVC insertion. Major components of maintenance bundles included hand hygiene; hub/connector/port disinfection; dressing replacement; clean and dry state of insertion site; and removal of blood clot at hub/connector/port.

A three-level inspector mechanism, called "Internal cross-check, Supervision of part-time IPC professionals, and External feedback from full-time IPC professionals", was established to ensure that cross-check work was subject to efficient quality control. First, if executor failed to correct the violation after the checker identified a violation, the checker should page ICU part-time IPC professionals who had been authorized by the infection management department to stop the procedure. Second, part-time IPC professionals carried out a weekly follow-up spot check on the completion of the internal cross-check. Then, the full-time IPC professionals gave results feedback once a quarter and put forward suggestions for continuous improvement.

### Central venous catheters

Identical double-/triple- lumen Certofix® central venous catheters (Opaque catheter made of polyurethane with Soft-tip, Safsite® valves, Markings to verify position, Colour-coded Luer Lock connections, Fixation wing, Connecting-cable and Clip, 20 mm Lumen, B. Braun Melsungen AG, Melsungen City, Germany) were used in all patients. Catheters were inserted using the Seldinger technique and full aseptic technique by a physician who had been granted the insertion qualification by the medical department. I.V. transparent film dressing with border (3 M Tegaderm™, Neuss, Germany) was placed over the exit site at the time of insertion.

### Definitions and data collection

Primary endpoint was CABSI followed by Centers for Disease Control and Prevention (CDC)/National Healthcare Safety Network (NHSN) definitions and criteria (NHSN Bloodstream Infection Event: https://www.cdc.gov/nhsn/pdfs/pscmanual/4psc_clabscurrent.pdf:) [16]. CABSI was defined as a laboratory-confirmed primary BSI that developed in patients wherein an eligible bloodstream infection-causing organism was identified and a CVC was placed at least 2 calendar days prior to the infection onset.

An independent CABSI prospective whistle-blower system, which was developed by the healthcare-associated infections quality control center in Sichuan province, was used to continuously monitor every case with CVC insertion. For each case, suspicious CABSI cases were automatically screened out by the intelligent identification program of the whistle-blower system if any of the following conditions were met: ① Fever (> 38 °C), ② Hypotension (systolic blood pressure < 90 mmHg and/or diastolic blood pressure < 60 mmHg), ③ Oliguria(< 400 ml/day), ④ Any microorganism cultured from ≥ 1 blood cultures, ⑤ CABSI cases that have been prospectively entered into the HAI electronic system by physicians.

To accomplish the study objectives, four infectious disease specialists revised the electronic medical records of all screened cases to check if all NHSN criteria were fulfilled.

In all cases the following variables were recorded: age, sex, hemodialysis, allogeneic blood transfusion (ABT), mechanical ventilation, urinary catheter insertion (UCI), tracheotomy, scheduled or non-scheduled surgery, reason for admission [International Classification of Diseases (ICD)-10 coded] [17], diabetes mellitus, hypertension, chronic obstructive pulmonary disease (COPD) and community infections.

### Statistical analysis

#### Randomization

Propensity score matching (PSM), a method of post-randomization, was performed between intervention and control groups using specific R Package Matching version 4.9–2 to optimize inter group comparability. To minimize the impact of potential bias, we performed the following analysis: 1. Standardized mean differences (SMD) were determined the baseline characteristics that lead to incompatibility between groups. And the SMD of less than 0.1 was considered as an indicator of good balance [18]. 2. A logistic regression model was used to calculate the propensity scores. 3. A k-nearest neighbor algorithm was used to make a 1:1 match without replacement using the caliper of width equal to 0.2 of the standard deviation of the legit of the propensity scores [19]. 4. SMD was used again to determine inter group comparability after PSM.

### Restricted cubic spline model

A multivariable logistic model with restricted cubic splines (RCS) was built using specific R Package Regression Modeling Strategies (RMS) version 6.2–0 to evaluate the impact of CVC-insertion in different sites on CABSI. RCS has been widely described as a valid strategy to realize the correlation analysis between continuous exposure and outcomes [20–22]. The spline was defined using five knots at the 5th, 25th, 50th, 75th, and 95th percentiles. The threshold was determined as the point in time with the smallest Odd Ratio (OR).

### Time-to-event analysis

The Kaplan–Meier survival functions were used to estimate the cumulative hazard of CABSI. Log rank (Mantel-Cox) test was used for time-to-event analysis. For infection-free survival analysis, survival endpoints in this study were 30 days from insertion until CABSI.

### Data analysis

R software (v3.6.1) under RStudio (v1.2.5001) was utilized for data analyses. Data are presented as number (corresponding percentage) for categorical variables, and as median (interquartile range, IQR) for non-normally continuous variables and as mean ± standard deviation for normally continuous variables. Using Wilcoxon rank sum test/one-way ANOVA for continuous variables and the χ^2^ test / Fischer’ exact test for categorical variables, data were compared between the two periods. Using specific R Package VIOPLOT version 0.3.7, Violin plots were used to visualize the incidence densities of CABSI between the two periods. All tests were 2-sided with an $\alpha$ level of 0.05.

## Results

**Fig. 1:**
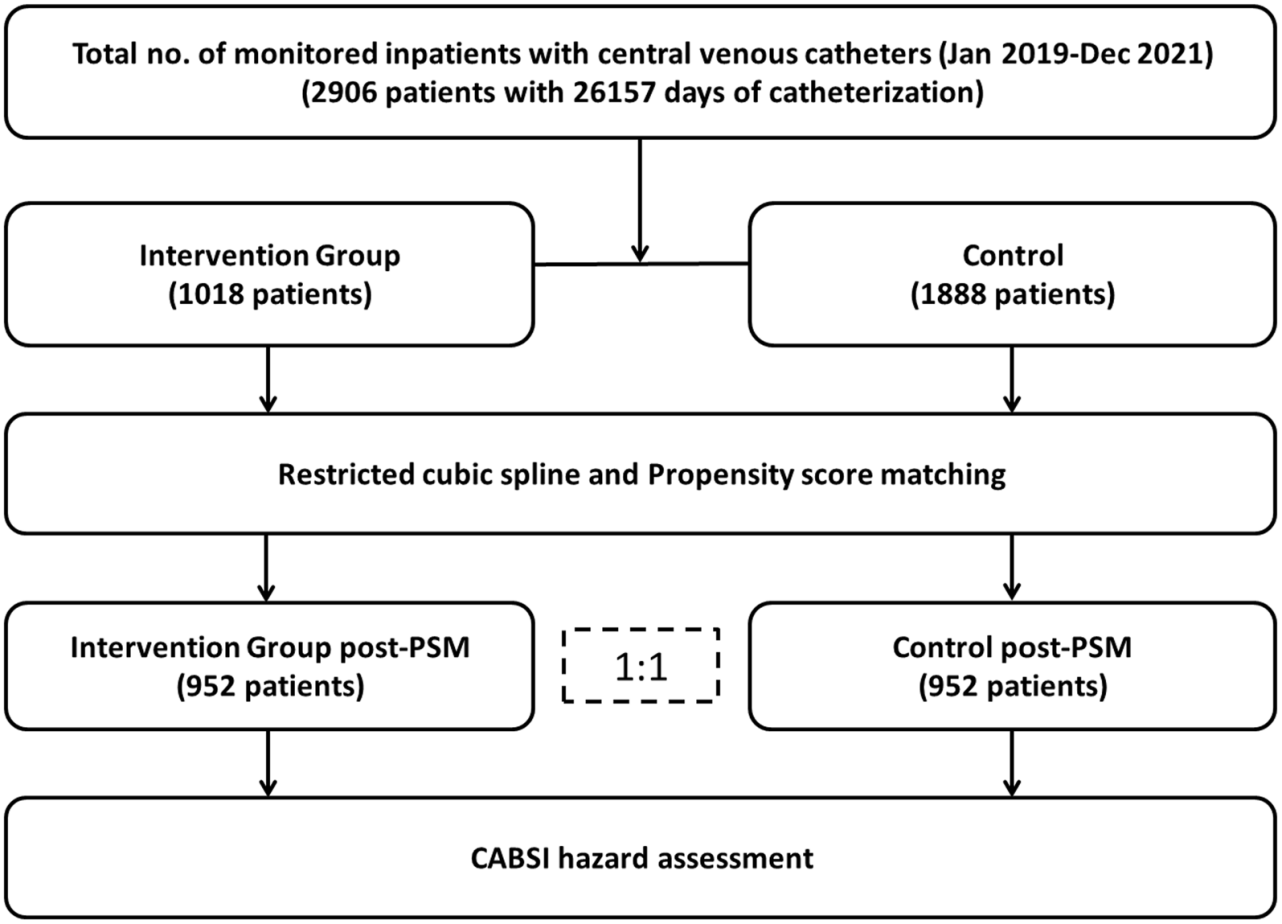
Cases selection algorithm for our study population. A total of 2906 patients with 26,157 CVC-days were included. CABSI, indicates Central Venous Catheter-associated Bloodstream Infection; PSM, Propensity Score Matching

During the study period, a total of 2906 patients with 26,157 CVC-days fulfilled the selection criteria (Period 1, 1888 patients with 17,007 CVC-days, and Period 2, 1018 patients with 9150 CVC-days, respectively; Fig. 1). As shown in Additional file 1: Table 3, patients with femoral (FEM)-vein catheterization were significantly younger (F = 5.000, *P* = 0.007), less community infection (χ^2^ = 27.451, *P* < 0.001), less ABT (χ^2^ = 16.914, *P* < 0.001), less mechanical ventilation (χ^2^ = 612.095, *P* < 0.001), less tracheotomy (χ^2^ = 11.540, *P* = 0.003) and less COPD (χ^2^ = 12.437, *P* = 0.002) than patients with subclavian (SC)-vein or jugular-vein catheterization. The density incidence of CABSI was statistically higher for femoral than for jugular (14.02 versus 7.76, *P* < 0.001) and subclavian (14.02 versus 5.25, *P* < 0.001) access.

**Fig. 2:**
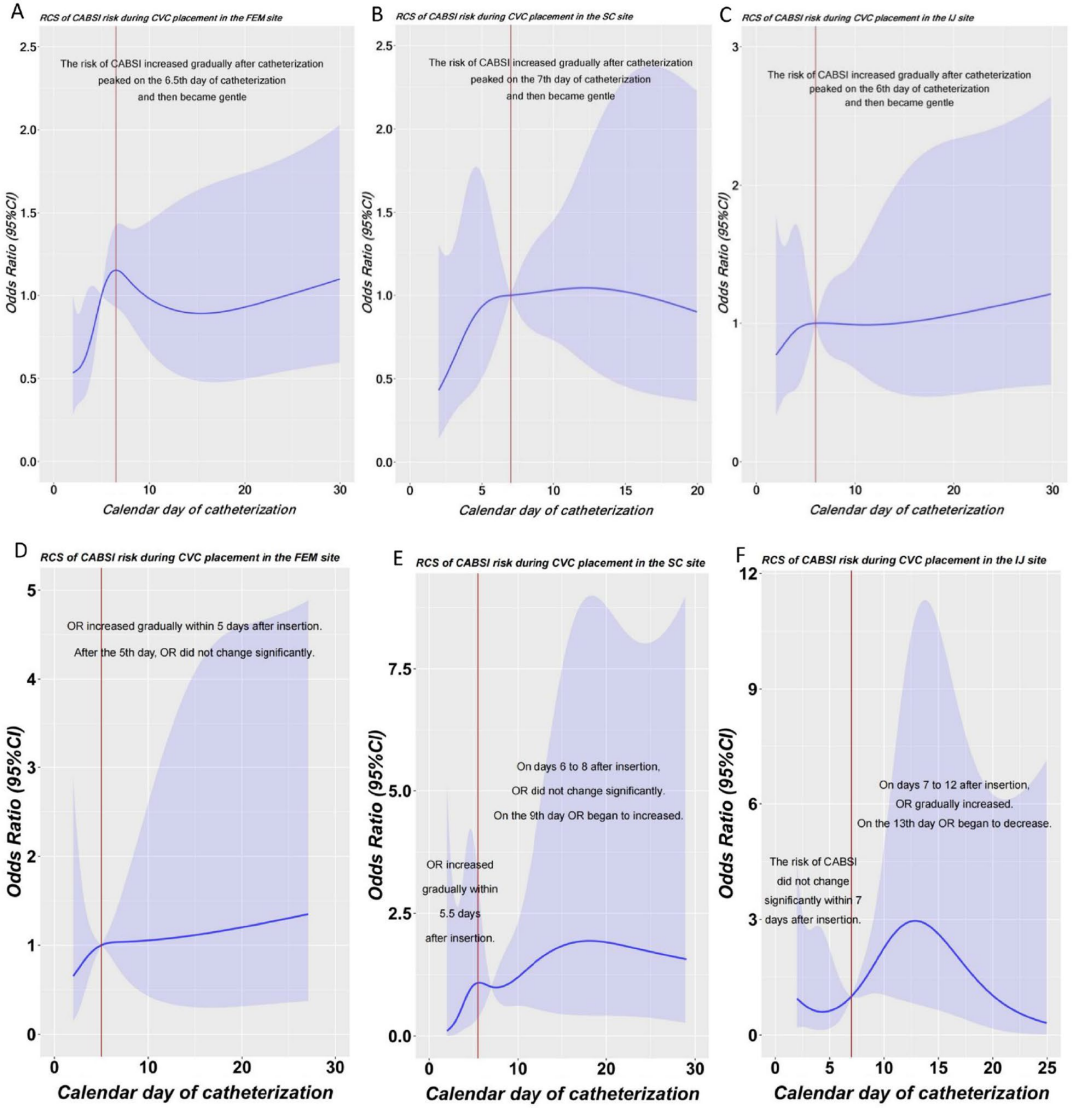
Restricted cubic spline of CABSI risk. **A** In period 1, OR value increased rapidly in the first 6.5 days of femoral-vein catheterization and became stable after the 6.5th day. **B** In period 1, OR value increased rapidly in the first 7 days of subclavian-vein catheterization and became stable after the 7th day. **C** In period 1, OR value increased rapidly in the first 6 days of jugular-vein catheterization and became stable after the 6th day. **D** In period 2, OR value increased rapidly in the first 5 days of femoral-vein catheterization and became stable after the 5th day. **E** In period 2, OR value increased rapidly in the first 5.5 days of subclavian-vein catheterization. On days 6 to 8 after insertion OR did not change significantly, and began to increase on the 9th day. **F** In period 2, OR value did not change significantly in the first 7 days of jugular-vein catheterization. On days 7 to 12 after insertion OR gradually increased, and began to decrease on the 13th day. RCS, indicates Restricted Cubic Spline; CABSI, entral Venous Catheter-associated Bloodstream Infection; FEM, Femoral; SC, Subclavian; IJ, Internal Jugular

Before intervention in Period 1, the result of the restricted cubic spline model is shown in Fig. 2. In the first 6.5 days of femoral-vein catheterization, the CABSI risk increased rapidly, and the OR value increased day by day. However, after the 6.5th day the OR value became stable (Fig. 2A). The CABSI risk increased rapidly in the first 7 days of subclavian-vein catheterization and became stable after the 7th day (Fig. 2B). The CABSI risk increased rapidly in the first 6 days of jugular-vein catheterization and became stable after the 6th day (Fig. 2C).

During the intervention period, performance of cross-check is shown in Tables 1, 2. The completion rate of nurses' check for insertion practice was 100.00% (1018/1018), while the completion rate of doctors' check for maintenance practice was 81.47% (2488/3054). In the results of nurses' check for insertion practice, the effective compliance rate of most items exceeded 80.00%, except for the item "Was the ultrasonic guidance used for internal jugular cather insertion?", which was only 56.19%. At the same time, because some nurses did not trace the past data, the item "Had daily skin cleaning with 2% chlorhexidine been performed?" had 80 missing values. In the results of doctors' check for maintenance practice, the effective compliance rate of most items exceeded 80.00%, except for the item "Was there any blood clot at the catheter hubs, needleless connectors, and injection ports?", which was only 78.18%. Because the doctors' check was not always at the time point of the maintenance process, some missing values inevitably appeared in this part of the results. After our evaluation, these missing values were considered to be unavoidable and conformed to the actual clinical state, and had little impact on the results.

**Table 1:** Comparison of clinical characteristics between the intervention and control groups

Variable	Period 1 (n = 1888)	Period 2 (n = 1018)	Statistic	*P *value
CABSIs	173 (9.16)	57 (5.60)	4.771*	0.029
Age, mean (SD),y	60.50 17.11)	61.66 (17.08)	-1.745†	0.081
Male	1227 (64.99)	658 (64.64)	0.036*	0.849
*Department*				
Surgical-ICU	492(26.06)	305(29.96)	83.981*	< 0.001
Emergency-ICU	876(46.40)	369(36.25)		
Medical-ICU	259(13.72)	250(24.56)		
Geriatrics-ICU	125(6.62)	39(3.83)		
Neurology-ICU	23(1.22)	20(1.96)		
Neurosurgical-ICU	113(5.99)	35(3.44)		
*CVC insertion sites*				
Femoral	721(38.19)	302(29.67)	42.854*	< 0.001
Subclavian	551(29.18)	416(40.86)		
Internal jugular	676(32.63)	300(29.47)		
Community Infections	476(25.21)	254(24.95)	0.024*	0.877
Scheduled or Non-scheduled Surgery	1776(94.07)	942(92.53)	2.570*	0.109
Allogeneic Blood Transfusion	1203(63.72)	689(67.68)	4.573*	0.032
Urinary Catheter Insertion	1433(75.90)	729(71.61)	6.389*	0.011
Hemodialysis	21(1.11)	10(0.98)	0.106*	0.745
Mechanical Ventilation	1284(68.01)	658(64.64)	3.392*	0.066
Tracheotomy	191(10.12)	109(10.71)	0.249*	0.618
Hypertension	612(32.45)	338(33.20)	0.186*	0.666
Diabetes mellitus	384(20.29)	231(22.69)	2.194*	0.139
Chronic Obstructive Pulmonary Disease	227(12.02)	108(10.61)	1.297*	0.255
*Principal diagnosis*				
Certain infectious diseases and parasites	89(4.71)	92(9.04)	60.749*	< 0.001
Tumor	110(5.83)	73(7.17)		
Blood and hematopoietic diseases and certain diseases involving immune mechanisms	7(0.37)	1(0.10)		
Endocrine, nutritional, and metabolic diseases	37(1.96)	26(2.55)		
Mental and behavioral disorders	8(0.42)	4(0.39)		
Nervous system diseases	31(1.64)	26(2.55)		
Circulatory diseases	370(19.60)	182(17.88)		
Respiratory diseases	469(24.84)	221(21.71)		
Digestive diseases	309(16.37)	217(21.32)		
Skin and subcutaneous tissue diseases	4(0.21)	5(0.49)		
Musculoskeletal system and connective tissue diseases	15(0.79)	3(0.29)		
Genitourinary diseases	66(3.50)	29(2.85)		
Pregnancy, childbirth, and puerperium	8(0.42)	5(0.49)		
Congenital malformations, deformation, and chromosomal abnormalities	6(0.32)	2(0.20)		
Abnormal symptoms, signs, clinical and laboratory results, and cannot be classified in other categories	20(1.06)	5(0.496)		
Injury, poisoning and other external pathogenic factors	337(17.85)	126(12.38)		
External causes of illness and death	2(0.11)	1(0.10)		

*Pearson's chi-squared test

^†^Student t-test

**Table 2:** Performance of cross check in intervention period

Checklist items	Execution (no.)	Missing (no.)	Effective compliance rate (%)
*Checklist for CVC insertion*			
1. Was the inserter approved by the medical department for insertion qualification?	1018	0	100.00%
2. Had daily skin cleaning with 2% chlorhexidine been performed?	782	80	83.37%
3. Whether hand hygiene was performed before insertion?	1018	0	100.00%
4. Were all items required for intravenous insertion prepared in advance?	902	0	88.61%
5. Was the maximum sterile barrier precautions performed?	1000	0	98.23%
6. Was the patient to be covered with a full-body sterile drape?	1000	0	98.23%
7. Skin preparation?	1018	0	100.00%
8. Was the ultrasound guidance used for internal jugular catheter insertion?	572	0	56.19%
9. Whether hand hygiene was performed at insertion?	991	0	97.35%
10. Whether the aseptic operation procedures were broken?	1018	0	100.00%
11. Was the correct dressing used?	1018	0	100.00%
12. Was hand hygiene performed after taking off gloves?	1018	0	100.00%
*Checklist for CVC maintenance*			
1. Whether hand hygiene was performed?	1286	1144	95.76%
2. Whether the dressing became damp, loosened, or visibly soiled?	2202	0	88.50%
3. Whether the catheter-insertion sites were clean or dry?	2088	0	83.92%
4. Was there any blood clot at the catheter hubs, needleless connectors, and injection ports?	1945	0	78.18%
5. Whether an alcoholic chlorhexidine preparation, 70% alcohol, or povidone-iodine was used to disinfect catheter hubs, needleless connectors, and injection ports before accessing the catheter?	1515	973	100.00%
6. Whether mechanical friction was applied for not less than 5 s to reduce pollution during hub/connector/port disinfection?	1515	973	100.00%

**Fig. 3:**
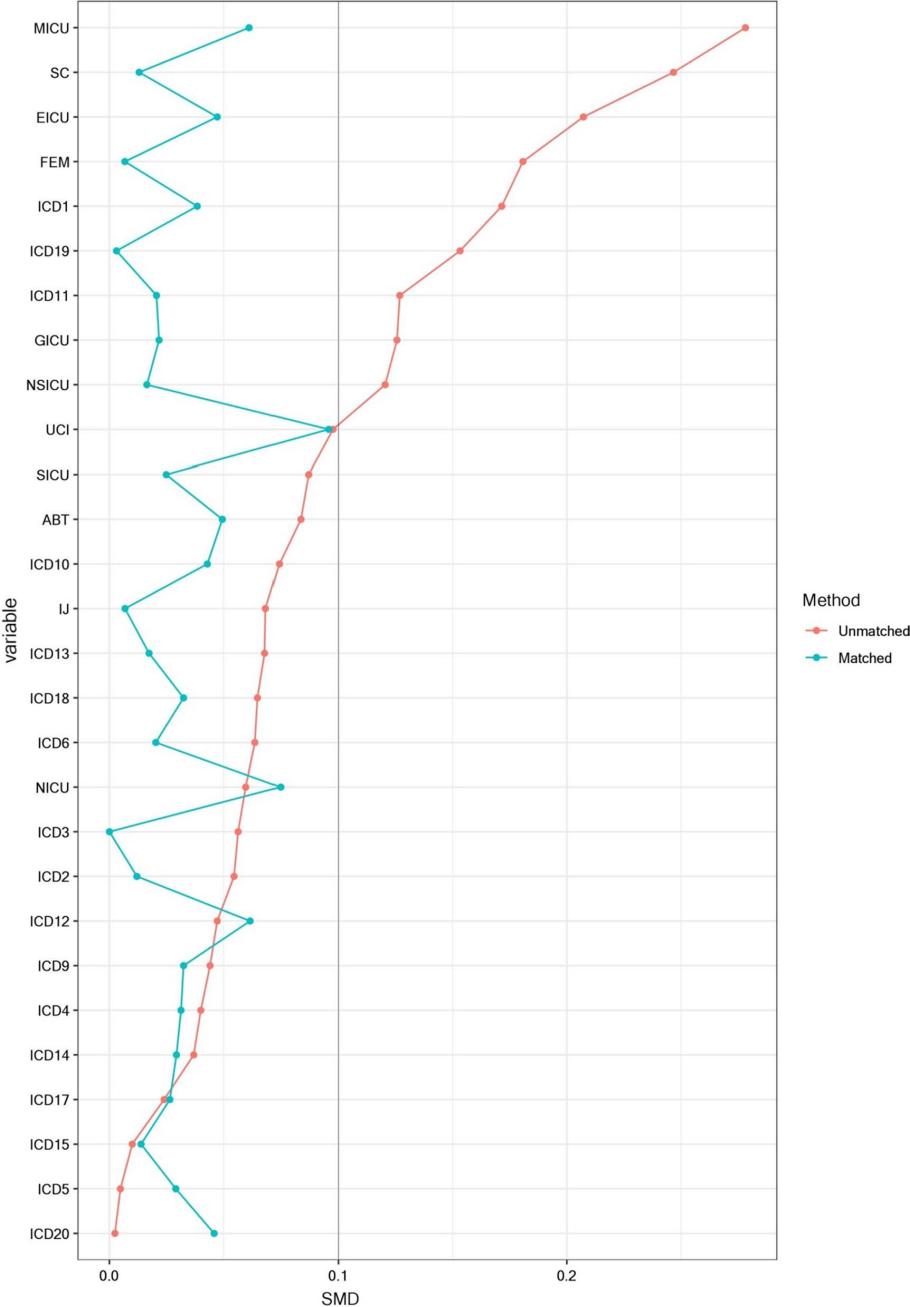
Standardized mean differences (SMD) before and after matching. PSM improved the balance on the investigated baseline characteristics with all SMD between groups decreasing to 0.1. SMD, indicates Standardized Mean Differences; ABT, Allogeneic Blood Transfusion; UCI, Urinary Catheter Insertion; SICU, Surgical-ICU; EICU, Emergency-ICU; MICU, Medical-ICU; NICU, Neurology-ICU; NSICU, Neurosurgical-ICU; GICU, Geriatrics-ICU; FEM, Femoral; SC, Subclavian; IJ, Internal jugular; ICD, International Classification of Diseases

After intervention in Period 2, the acute period of rapid increase in CABSI risk was shortened, 6.5 to 5 days for femoral-vein catheterization (Fig. 2D), and 7 to 5.5 days for subclavian-vein catheterization (Fig. 2E), respectively. For jugular-vein catheterization, the acute onset period after insertion disappeared (Fig. 2F). However, after the end of the 7-day intervention, the CABSI risk in subclavian-vein and jugular-vein catheterization increased inversely (Fig. 3B and C).

Before PSM, the density incidence of CABSI decreased from 10.17 to 6.23/1,000 CVC-days (*P* < 0.001) between Periods 1 and 2. As noted in Table 1, there were significant differences in three basic characteristics between Period 1 and 2, including admitted ICU (χ^2^ = 83.981, *P* < 0.001), CVC insertion sites (χ^2^ = 42.854, *P* < 0.001), ABT (χ^2^ = 4.573, *P* = 0.032), UCI (χ^2^ = 6.389, *P* = 0.011) and principal diagnosis (χ^2^ = 60.749, *P* < 0.001). PSM improved the balance on the baseline characteristics with all SMD decreasing to 0.1 between groups (Fig. 3).

**Fig. 4:**
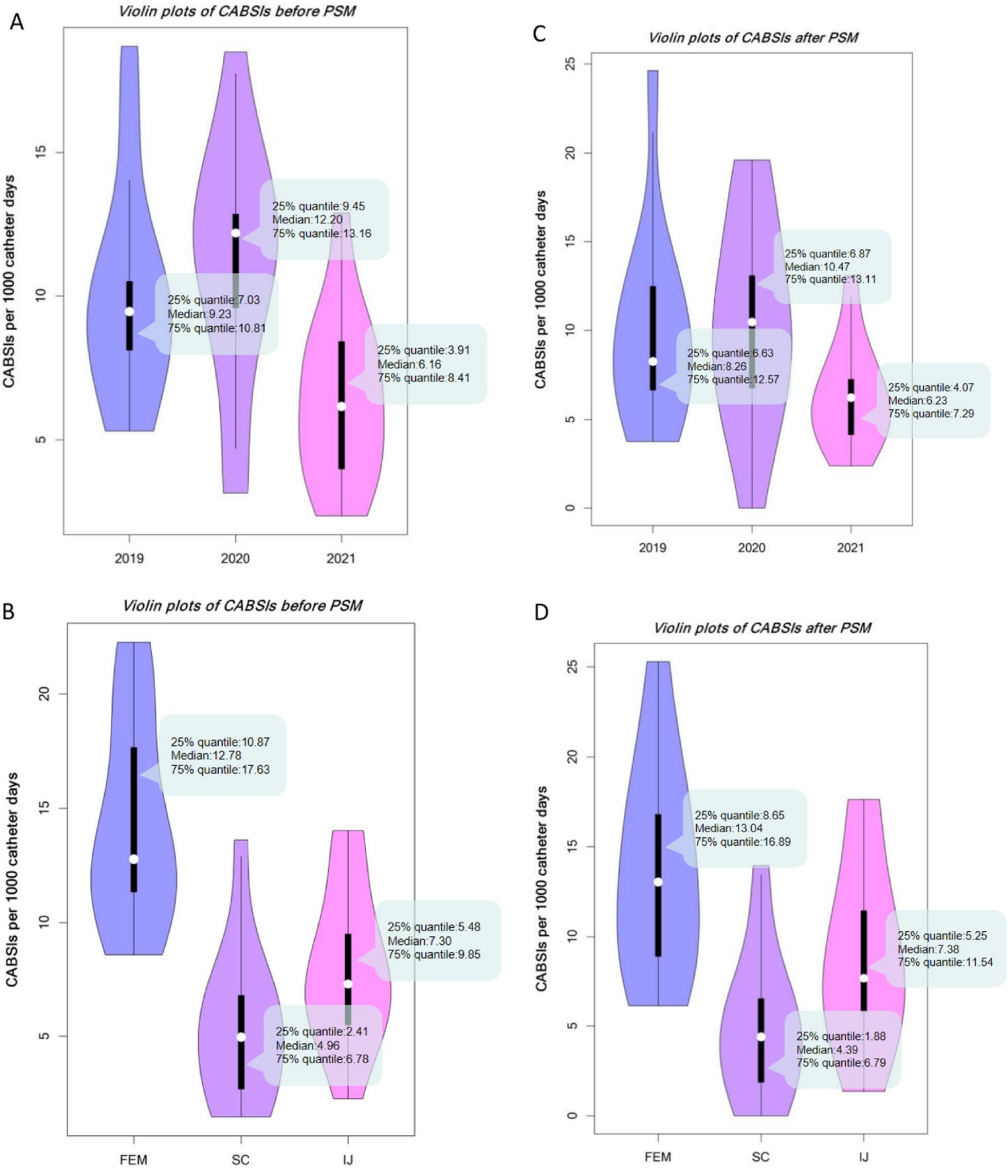
Violin plots of CABSI before and after matching. **A** The density incidence of CABSI was significantly reduced in 2021 compared to 2019 and 2020 before PSM. **B** The FEM site was associated with higher density incidence of CABSI compared to the SC site and the IJ site before PSM. **C** The density incidence of CABSI was also significantly reduced in 2021 compared to 2019 and 2020 after PSM. **D** The FEM site was also associated with higher density incidence of CABSI compared to the SC site and the IJ site after PSM. CABSI, indicates Central Venous Catheter-associated Bloodstream Infection; PSM, Propensity Score Matching; FEM, Femoral; SC, Subclavian; IJ, Internal Jugular

**Fig. 5:**
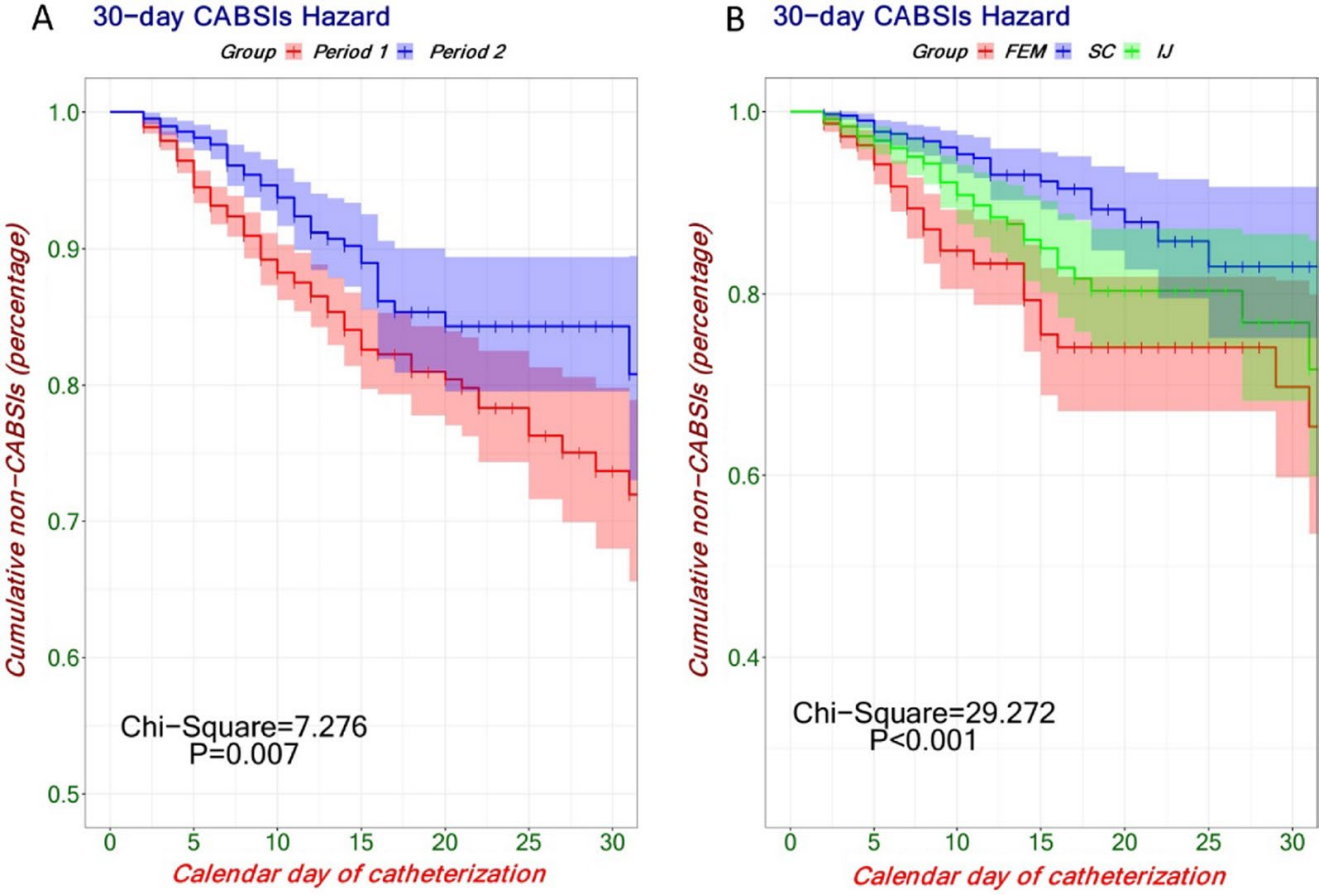
30-day CABSI hazard after propensity score matching. **A** Times to 30-day CABSI were significantly decreased in Period 2 compared to Period 1. **B** The FEM site was associated with higher CABSI cumulative hazard compared to the SC site and the IJ site. CABSI, indicates Central Venous Catheter-associated Bloodstream Infection; FEM, Femoral; SC, Subclavian; IJ, Internal Jugular

After PSM, the final analyzed data consisted of 952 patients with 8792 CVC-days in Period 1 and 952 patients with 8537 CVC-days in Period 2 (Fig. 1). The incidence and density incidence of CABSI decreased from 9.45% to 5.67% (*P* = 0.002) and from 10.24 to 6.33/1,000 CVC-days (*P* < 0.001) between Periods 1 and 2, respectively. Figure 4C shows that the incidence and density incidence of CABSI was significantly reduced in Period 2 compared to Period 1 after adjustment by PSM. The differences remained significant in unadjusted analyses (Fig. 4A). No matter whether the analysis was adjusted by PSM or not, the FEM site was associated with higher density incidence of CABSI compared to the SC site and the IJ site (Fig. 4B and Fig. 4D). Times to 30-day CABSI were also significantly decreased in Period 2 compared to Period 1 (χ^2^ = 7.276, *P* = 0.007, Fig. 5A). The FEM site was associated with higher CABSI cumulative hazard compared to the SC site and the IJ site (χ^2^ = 29.272, *P* < 0.001, Fig. 5B).

## Discussion

In this prospective, randomized, before–after real-world study in critically ill patients, we showed that the implementation of enhanced check during the first 7 calendar days after CVC insertion could reduce the CABSI risk. To our knowledge, this is the first report on CABSI prevention based on the acute phase. Taking the acute phase of CABSI as a window of opportunity and relying on enhanced check to ensure adherence to infection prevention practices, a significant effect on the overall CABSI risk and shortened duration of acute onset period were observed.

Developing a supervise process, through the use of a checklist, is an effective measure to ensure adherence to optimal CABSI prevention practices. Significant effect on reducing the CABSI risk has long been confirmed in the use of a CVC insertion checklist, as others suggested [23, 24], but also in the use of a CVC maintenance checklist as our study found. Unfortunately, evidence-based recommendations on CVC maintenance checklist are rare at present [11, 25]. In our opinion, a check process for CVC maintenance is indispensable for the following 2 reasons: (1) significantly reduced overall CABSI risk; and (2) reverse growth of CABSI risk after the end of the 7-day intervention. The ongoing need for enhanced check is supported by these findings. Of note, the cross-check model also avoids the subjective barriers of physicians and nurses mentioned by Berenholtz and coworkers [15]. Physicians and nurses perceived that they need to work together to ensure the CABSI prevention in the whole process of CVC insertion and maintenance, because they were both executors of specific practices and checkers at different stages. When presented in this light, physicians would not perceive their credibility and authority were challenged if they were corrected by nursing staff, and nurses were also activated to perform check work with the participation of physicians in the CVC maintenance. Furthermore, the reverse growth of CABSI risk after intervention suggests that the existence of considerable Hawthorne Effect, which has been used in psychology for several decades as a strategy for health-related behaviors [26], can be regarded as a decisive dynamic momentum to ensure adherence to optimal CABSI prevention practices continuously. Importantly, the use of a checklist, as a low-cost and high-yield intervention, was easier to implement because of rare obstacles. Correspondingly, some expensive interventions such as the use of chlorhexidine-containing dressings, even if it had been considered as an “essential practice” in the recently updated guideline [27], were difficult to popularize under the current medical insurance system in China due to their high acquisition costs and limited information regarding cost-effectiveness [28].

Similar to other infectious diseases, CABSI has acute phase characteristics [29, 30]. The restricted cubic spline illustrated that the CABSI risk was significantly increased within the first 7 days after CVC insertion, and was relatively flat after 7 days. The acute phase, defined as 7 days after CVC insertion, was confirmed by this study as a window of opportunity for reducing CABSI risk. Buetti and coworkers reported identical results in the CABSI risk of short-term dialysis catheters, and suggested that the targeted prevention strategies should focus on the first week after the catheter insertion [31]. Without new measures beyond guidelines recommendations in this study, the targeted strategy of enhanced check has successfully reduced the CABSI risk and shorten the acute phase duration. As far as we know, the present study is the first report demonstrating that a prevention practice was associated with the shortened duration of the CABSI acute phase. Consider, too, that the effect of reducing CABSI rate is partly due to the reduced odds ratio, and partly due to the shortened acute phase that has not yet attracted sufficient attention. The shortened acute phase can be used as a potential indicator to evaluate the effectiveness of CABSI prevention practice in future study. Importantly, in the case of scarce health-related resources, a targeted CABSI prevention strategy based on the acute phase in low-income and middle-income countries is, predictably, of great help to achieve the best cost-effectiveness.

We conducted a propensity score matching to avoid the 2 major bias that commonly lead to an inter-group imbalance in the large sample studies: (1) different baseline clinical characteristics, including Allogeneic Blood Transfusion, Urinary Catheter Insertion, ICU Department, and Principal Diagnosis, were the main reason for poor comparability between groups; and (2) the choice of insertion site significantly influence the incidence of CABSI. Randomized studies have demonstrated excess risk of CABSI associated with the femoral venous catheterization compared wlith the subclavian catheterization or the internal jugular catheterization in ICU patients [32–34]. Most notably, the specific independent effects of different insertion sites could be produced by a same intervention. We found that the effect reversal after intervention was more likely to occur in the catheterization at internal jugular or subclavian sites than that at femoral sites. In view of the current guideline-based practices have targeted the whole CVC without sufficiently distinguishing different insertion sites in detail, future studies need to do more in terms of possible specific independent effects.

The CABSI prospective whistle-blower system, which had been already certified by the National Copyright Administration of the People's Republic of China (Additional file 1: Fig 1), served as a powerful screening tool for potential cases of CABSI in this study. Like Nosocomial Infection Marker and Trick's computer algorithms [35–37], this system reduced a lot of manual effort and would allow IPC professionals to focus on other prevention interventions. The original characteristics of this system were to add detection of clinical symptoms rather than rely solely on microbiology data. Adding would, accordingly, allow IPC professionals to remove false-whistled cases more effectively and forestall partially missed diagnosis due to low prevalence of positive blood cultures [38].

A number of limitations of this study should be considered. First, we evaluated interventions in an academic medical center with a relatively high incidence of CABSI, and as a result, it was difficult to judge whether our interventions equally effective in the case of low CABSI rate or not. Rather, we might still underestimate our CABSI rate because participants with only one catheter were included in this study, potentially limiting the ability to generalize. Nevertheless, our interventions were significantly effective and were cost-effective and, therefore, can be widely used.

Second, the before-after study design have not accounted for other confounding factors that may have led to imbalance between groups over time. For example, standardized measures of patient severity of illness, which generally include Acute Physiology and Chronic Health Evaluation, Sequential Organ Failure Assessment, Charlson Comorbidity Index and Glasgow Coma Scale, were not applied due to the data blackout caused by different management styles during the study period, as mentioned by our team member Huang and coworkers in November 2019 [39]. Nevertheless, severity of illness tends to change little over time as previously demonstrated by Pronovost et al.[40] and, therefore, has little impact on our results.

## Conclusion

In summary, the implementation of enhanced check during the first 7 calendar days after CVC insertion shortens the duration of the CABSI acute phase and tends to decrease CABSI rate.

## Supplementary Information

**Additional file 1.** Necessary accessory materials: Checklist for CVC insertion and maintenance, Certificate issued by the National Copyright Administration of the People's Republic of China, and Baseline characteristics according to catheter site.

## Data Availability

The datasets used and analysed during the current study are available from the corresponding author on reasonable request.

## References

[1] European Centre for Disease Prevention and Control. Healthcare-associated infections acquired in intensive care units. In: ECDC. Annual epidemiological report for 2017. Stockholm: ECDC; 2019. https://www.ecdc.europa.eu/sites/default/files/documents/AER_for_2017-HAI.pdf Accessed June 1, 2022.

[2] Takashima M, Schults J, Mihala G (2018). Complication and failures of central vascular access device in adult critical care settings. Crit Care Med.

[3] Ullman AJ, Marsh N, Mihala G (2015). Complications of central venous access devices: a systematic review. Pediatrics.

[4] Ista E, van der Hoven B, Kornelisse RF (2016). Effectiveness of insertion and maintenance bundles to prevent central-line-associated bloodstream infections in critically ill patients of all ages: a systematic review and meta-analysis. Lancet Infect Dis.

[5] Rosenthal VD, Maki DG, Mehta Y (2014). International nosocomial infection control consortium (INICC) report, data summary of 43 countries for 2007–2012. Device associated module. Am J Infect Control.

[6] National Health Commission of the People`s Republic of China. National report on the services, quality and safety in medical care system (2019). Science and Technology Literature Press, Beijing

[7] Milstone AM, Reich NG, Advani S (2013). Catheter dwell time and CLABSIs in neonates with PICCs: a multicenter cohort study. Pediatrics.

[8] Voets PJGM (2018). Central line-associated bloodstream infections and catheter dwell-time: a theoretical foundation for a rule of thumb. J Theor Biol.

[9] Lucet JC, Bouadma L, Zahar JR (2010). Infectious risk associated with arterial catheters compared with central venous catheters. Crit Care Med.

[10] Timsit JF (2000). Scheduled replacement of central venous catheters is not necessary. Infect Control Hosp Epidemiol.

[11] Marschall J, Mermel LA, Fakih M (2014). Strategies to prevent central line-associated bloodstream infections in acute care hospitals: 2014 update. Infect Control Hosp Epidemiol.

[12] National Health Commission of the People's Republic of China. Guidelines for the prevention and control of vascular catheter related infections (2021 Edition). Infect Dis Info, 2021;34(4):289–295.

[13] Lv Y, Huang X, Lan Y, et al. Peripherally inserted central catheters have a protective role and the effect of fluctuation curve feature in the risk of bloodstream infection compared with central venous catheters: a propensity-adjusted analysis. BMC Infect Dis. 2022;22(1):289. Published 2022 Mar 26. 10.1186/s12879-022-07265-x 10.1186/s12879-022-07265-x PMC8961920 35346073

[14] Ider BE, Adams J, Morton A (2012). Using a checklist to identify barriers to compliance with evidence-based guidelines for central line management: a mixed methods study in Mongolia. Int J Infect Dis.

[15] Berenholtz SM, Pronovost PJ, Lipsett PA (2004). Eliminating catheter-related bloodstream infections in the intensive care unit. Crit Care Med.

[16] Bloodstream Infection Event (Central Line-Associated Bloodstream Infection and non-central line-associated Bloodstream Infection)-Device associated module. Updated January 2022. https://www.cdc.gov/nhsn/pdfs/pscmanual/4psc_clabscurrent.pdf

[17] Meng Q, Liu A (2017). Guidelines for national disease classification and code application.

[18] Austin PC (2009). Using the standardized difference to ompare the prevalence of a binary variable etween two groups in observational research. Commun Stat Simul Comput.

[19] Austin PC (2011). Optimal caliper widths for propensity-score matching when estimating differences in means and differences in proportions in observational studies. Pharm Stat.

[20] Yamakawa K, Gando S, Ogura H (2019). Identifying sepsis populations benefitting from anticoagulant therapy: a prospective cohort study incorporating a restricted cubic spline regression model. Thromb Haemost.

[21] Navaratnam AV, Gray WK, Day J (2021). Patient factors and temporal trends associated with COVID-19 in-hospital mortality in England: an observational study using administrative data. Lancet Respir Med.

[22] Salazar MC, Rosen JE, Wang Z (2017). Association of delayed adjuvant chemotherapy with survival after lung cancer surgery. JAMA Oncol.

[23] Gozu A, Clay C, Younus F (2011). Hospital-wide reduction in central line-associated bloodstream infections: a tale of two small community hospitals. Infect Control Hosp Epidemiol.

[24] Sagana R, Hyzy RC (2013). Achieving zero central line-associated bloodstream infection rates in your intensive care unit. Crit Care Clin.

[25] Miller MR, Niedner MF, Huskins WC (2011). Reducing PICU central line-associated bloodstream infections: 3-year results. Pediatrics.

[26] Chen LF, Vander Weg MW, Hofmann DA, Reisinger HS (2015). The hawthorne effect in infection prevention and epidemiology. Infect Control Hosp Epidemiol.

[27] Buetti N, Marschall J, Drees M, et al. Strategies to prevent central line-associated bloodstream infections in acute-care hospitals: 2022 Update [published online ahead of print. Infect Control Hosp Epidemiol. 2022;43(5):1–17. 10.1017/ice.2022.87 10.1017/ice.2022.87 PMC9096710 35437133

[28] Heimann SM, Biehl LM, Vehreschild JJ (2018). Chlorhexidine-containing dressings in the prevention of central venous catheter-related bloodstream infections: a cost and resource utilization analysis. Am J Infect Control.

[29] Delaroche L, Bertine M, Oger P (2021). Evaluation of SARS-CoV-2 in semen, seminal plasma, and spermatozoa pellet of COVID-19 patients in the acute stage of infection. PLoS One..

[30] Arunkumar G, Devadiga S, McElroy AK (2019). Adaptive immune responses in humans during nipah virus acute and convalescent phases of infection. Clin Infect Dis.

[31] Buetti N, Ruckly S, Lucet JC (2019). Short-term dialysis catheter versus central venous catheter infections in ICU patients: a post hoc analysis of individual data of 4 multi-centric randomized trials. Intensive Care Med.

[32] Parienti JJ, Mongardon N, Mégarbane B (2015). Intravascular complications of central venous catheterization by insertion site. N Engl J Med.

[33] Merrer J, De Jonghe B, Golliot F (2001). Complications of femoral and subclavian venous catheterization in critically ill patients: a randomized controlled trial. JAMA.

[34] Buetti N, Ruckly S, Lucet JC (2020). The insertion site should be considered for the empirical therapy of short-term central venous and arterial catheter-related infections. Crit Care Med.

[35] Brossette SE, Hacek DM, Gavin PJ (2006). A laboratory-based, hospital-wide, electronic marker for nosocomial infection: the future of infection control surveillance?. Am J Clin Pathol.

[36] Ridgway JP, Sun X, Tabak YP (2016). Performance characteristics and associated outcomes for an automated surveillance tool for bloodstream infection. Am J Infect Control.

[37] Trick WE, Zagorski BM, Tokars JI (2004). Computer algorithms to detect bloodstream infections. Emerg Infect Dis.

[38] Clancy CJ, Nguyen MH (2013). Finding the "missing 50%" of invasive candidiasis: how nonculture diagnostics will improve understanding of disease spectrum and transform patient care. Clin Infect Dis.

[39] Xiaoqin Z, Qian W, Xiaoxiu L (2019). Prognostic value of Charlson weighted index of comorbidities combined sequential organ failure assessment score and procalcitonin in patients with sepsis. Chin Crit Care Med.

[40] Pronovost PJ, Angus DC, Dorman T (2002). Physician staffing patterns and clinical outcomes in critically ill patients: A systematic review. JAMA.

