# Supplementary material for: Effectiveness of enhanced check during acute phase to reduce central venous catheters-associated bloodstream infections: a before-after, real-world study
Source: Antimicrob Resist Infect Control. 2022 Dec 6;11:151. doi: 10.1186/s13756-022-01190-z (PMC9724293; doi:10.1186/s13756-022-01190-z)
Supplement: Supplementary file 1 — Additional file 1. Necessary accessory materials: Checklist for CVC insertion and maintenance, Certificate issued by the National Copyright Administration of the People's Republic of China, and Baseline characteristics according to catheter site. [file 13756_2022_1190_MOESM1_ESM.docx]

**eTable 1-Checklist for CVC insertion**

(For nurses)

| **Basic information** | | | | | |
| --- | --- | --- | --- | --- | --- |
| ICU: | Date and time: | Maintainer: | Inspector: | | |
| Inpatient: | Inpatient ID: | Sex: | Age: | | |
| Catheter-insertion site: | | □Emergency-insertion  □First selective-insertion  □Repeated selective-insertion | | | |
| **Preparation before insertion** | | | | | |
| 1. Was the inserter approved by the medical department for insertion qualification? | | | | | □ yes □no |
| 2. Had daily skin cleaning with 2% chlorhexidine been performed? | | | | | □ yes □no |
| 3. Whether hand hygiene was performed before insertion? | | | | | □ yes □no |
| 4. Were all items required for intravenous insertion prepared in advance?  Notes: Items that must be prepared:  □Intravenous insertion kit, □Catheter, □Sterile hole towel capable of covering the full-body,  □Skin disinfectant (alcoholic chlorhexidine solution containing more than 0.5% chlorhexidine),  □Disinfectant for hand hygiene, □Sterile gauze and sterile, transparent, semipermeable dressing. | | | | | □ yes □no |
| **At insertion** | | | | | |
| 5. Was the maximum sterile barrier precautions performed?  □Mask □Cap □Sterile gown □Sterile gloves | | | | □ yes □no | |
| 6. Was the patient to be covered with a full-body sterile drape? | | | | □ yes □no | |
| 7. Skin preparation  □Apply an alcoholic chlorhexidine solution containing more than 0.5% CHG,  □With the puncture as the center, the disinfection diameter ＞15cm, disinfection ≥2 times,  □The antiseptic solution must be allowed to dry before making the skin puncture. | | | | □ yes □no | |
| 8. Was the ultrasound guidance used for internal jugular catheter insertion? | | | | □ yes □no | |
| 9. Whether hand hygiene was performed at insertion? | | | | □ yes □no | |
| 10. Whether the aseptic operation procedures were broken? | | | | □ yes □no | |
| **After insertion** | | | | | |
| 11. Was the correct dressing used?  □Sterile, transparent, semipermeable dressings were routinely used,  □Use sterile gauze dressings for patients with fever, sweating, bleeding and/or oozing. | | | | □ yes □no | |
| 12. Was hand hygiene performed after taking off gloves? | | | | □ yes □no | |
| Notes: Every insertion of central venous catheters must be checked. If you have any questions, please call the Healthcare-associated Infection Control Center at 3275/ 4013.  Produced by Healthcare-associated Infection Control Center 2020.1 | | | | | |

**eTable 2-Checklist for CVC maintenance**

(For physicians)

| **Basic information** | | | | |
| --- | --- | --- | --- | --- |
| ICU: | Date and time: | Maintainer: | Inspector: | |
| Inpatient: | Inpatient ID: | Sex: | Age: | |
| Catheter-insertion site: | | | | |
| **Maintenance process** | | | | |
| 1. Whether hand hygiene was performed?  Notes: Any moment before and after palpating catheter insertion sites:  ①Flushing and/or locking lumen, ②Aspiration of blood, ③Medication with CVAD,  ④Dressing replacement, ⑤Extubation. | | | | □ yes □no |
| 2. Whether the dressing became damp, loosened, or visibly soiled? | | | | □ yes □no |
| 3. Whether the catheter-insertion sites were clean or dry? | | | | □ yes □no |
| 4. Was there any blood clot at the catheter hubs, needleless connectors, and injection ports? | | | | □ yes □no |
| 5. Whether an alcoholic chlorhexidine preparation, 70% alcohol, or povidone-iodine was used to disinfect catheter hubs, needleless connectors, and injection ports before accessing the catheter? | | | | □ yes □no |
| 6. Whether mechanical friction was applied for not less than 5 seconds to reduce pollution during hub/connector/port disinfection? | | | | □ yes □no |
| Notes: Three checks need to be completed within 7 days after central catheter insertion. The time point requirements for verification are the first day, the 4th day and the 7th day after insertion. If you have any questions, please call the Healthcare-associated Infection Control Center at 3275/ 4013.  Produced by Healthcare-associated Infection Control Center 2020.1 | | | | |

eFig 1


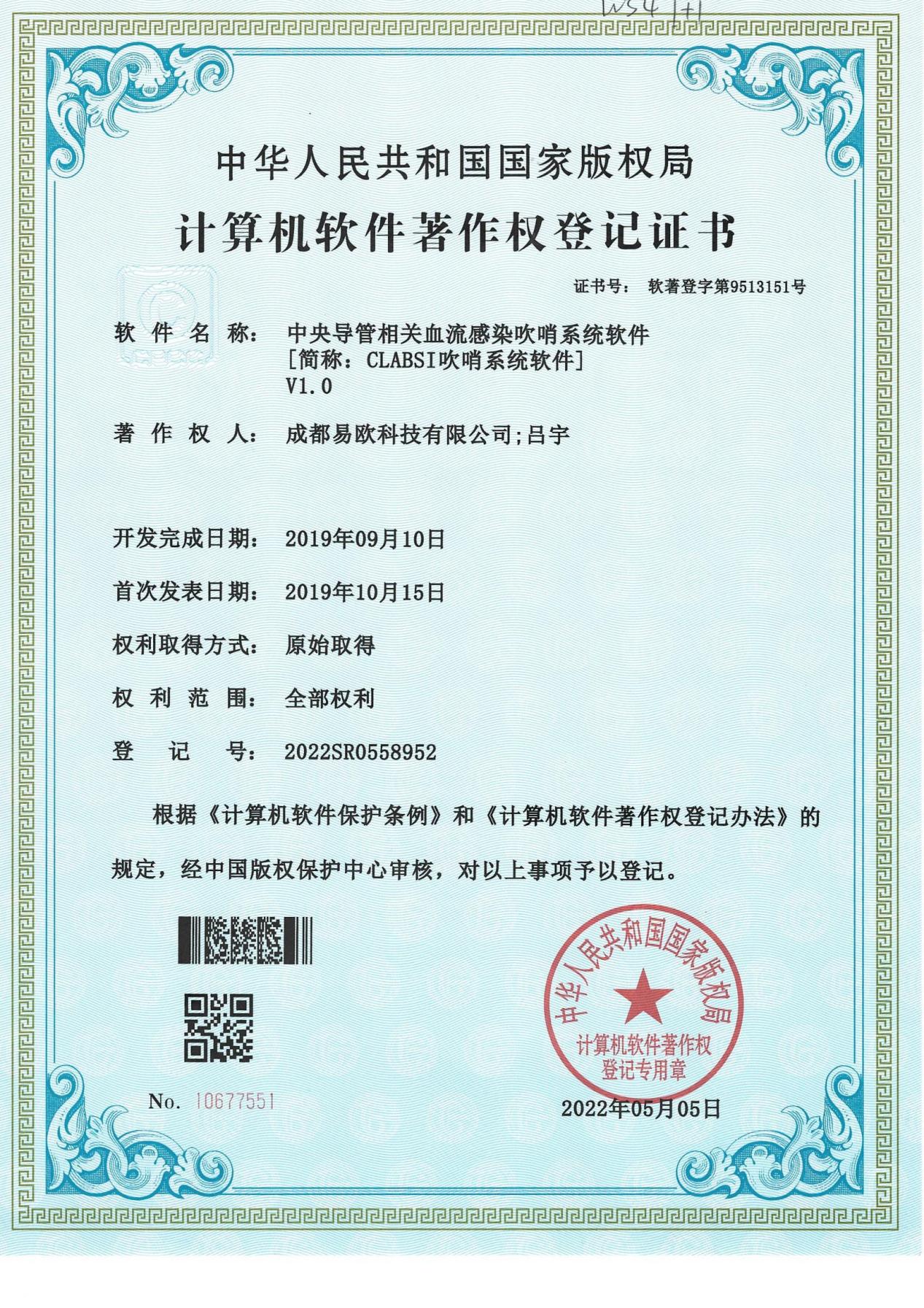


The CABSI prospective whistle-blower system had been already certified by the [National Copyright Administration of the People's Republic of China](http://dict.cn/National Copyright Administration of the People's Republic of China).

**eTable 3. Baseline characteristics according to catheter site**

|  | CVC Placement, No. (%) | | |  |  |
| --- | --- | --- | --- | --- | --- |
| Variable | FEM(n=1023) | SC(n=967) | IJ(n=916) | *Statistic* | *P-value* |
| Age, mean (SD),y | 59.58(17.80) | 61.91(17.06) | 61.32(16.27) | 5.000* | 0.007 |
| Male | 641(62.66) | 646(66.80) | 598(65.28) | 3.851† | 0.146 |
| Department |  |  |  | 778.504† | <0.001 |
| Surgical-ICU | 132(12.90) | 235(24.30) | 430(46.94) |  |  |
| Emergency-ICU | 734(71.75) | 298(30.82) | 213(23.25) |  |  |
| Medical-ICU | 72(7.04) | 307(31.75) | 130(14.19) |  |  |
| Geriatrics-ICU | 60(5.87) | 63(6.51) | 41(4.48) |  |  |
| Neurology-ICU | 12(1.17) | 18(1.86) | 13(1.42) |  |  |
| Neurosurgical-ICU | 13(1.27) | 46(4.76) | 89(9.72) |  |  |
| Community Infections | 211(20.63) | 297(30.71) | 222(24.24) | 27.451† | <0.001 |
| Scheduled or Non-scheduled Surgery | 962(94.04) | 899(92.97) | 857(93.56) | 0.941† | 0.625 |
| Allogeneic Blood Transfusion | 635(62.07) | 612(63.29) | 645(70.41) | 16.914† | <0.001 |
| Urinary Catheter Insertion | 746(72.92) | 736(76.11) | 680(74.24) | 2.672† | 0.263 |
| Hemodialysis | 12(1.17) | 7(0.72) | 12(1.31) | 1.700† | 0.427 |
| Mechanical Ventilation | 589(57.58) | 691(71.46) | 662(72.27) | 61.095† | <0.001 |
| Tracheotomy | 90(8.80) | 126(13.03) | 84(9.17) | 11.540† | 0.003 |
| Hypertension | 350(34.21) | 308(31.85) | 292(31.88) | 1.662† | 0.436 |
| Diabetes mellitus | 233(22.78) | 225(23.27) | 157(17.14) | 13.050† | 0.001 |
| Chronic Obstructive Pulmonary Disease | 101(9.87) | 140(14.48) | 94(10.26) | 12.437† | 0.002 |
| Principal diagnosis |  |  |  | 245.528† | <0.001 |
| Certain infectious diseases and parasites | 48(4.69) | 77(7.96) | 56(6.11) |  |  |
| Tumor | 39(3.81) | 45(4.65) | 99(10.81) |  |  |
| Blood and hematopoietic diseases and certain diseases involving immune mechanisms | 7(0.68) | 1(0.10) | 0(0.00) |  |  |
| Endocrine, nutritional, and metabolic diseases | 48(4.69) | 11(1.14) | 4(0.44) |  |  |
| Mental and behavioral disorders | 7(0.68) | 2(0.21) | 3(0.33) |  |  |
| Nervous system diseases | 21(2.05) | 22(2.28) | 14(1.53) |  |  |
| Circulatory diseases | 166(16.23) | 162(16.75) | 224(24.45) |  |  |
| Respiratory diseases | 201(19.65) | 304(31.44) | 185(20.20) |  |  |
| Digestive diseases | 194(18.96) | 171(17.68) | 161(17.58) |  |  |
| Skin and subcutaneous tissue diseases | 5(0.49) | 4(0.41) | 0(0.00) |  |  |
| Musculoskeletal system and connective tissue diseases | 11(1.08) | 4(0.41) | 3(0.33) |  |  |
| Genitourinary diseases | 65(6.35) | 15(1.55) | 15(1.64) |  |  |
| Pregnancy, childbirth, and puerperium | 2(0.20) | 2(0.21) | 9(0.98) |  |  |
| Congenital malformations, deformation, and chromosomal abnormalities | 2(0.20) | 2(0.21) | 4(0.44) |  |  |
| Abnormal symptoms, signs, clinical and laboratory results, and cannot be classified in other categories | 14(1.37) | 6(0.62) | 5(0.55) |  |  |
| Injury, poisoning and other external pathogenic factors | 191(18.67) | 139(14.37) | 133(14.52) |  |  |
| External causes of illness and death | 2(0.20) | 0(0.00) | 1(0.11) |  |  |

*Student t-test.

†Pearson's chi-squared test.
